# Targeting MMP-Regulation of Inflammation to Increase Metabolic Tolerance to COVID-19 Pathologies: A Hypothesis

**DOI:** 10.3390/biom11030390

**Published:** 2021-03-06

**Authors:** Eugenio Hardy, Carlos Fernandez-Patron

**Affiliations:** 1 Center for Molecular Immunology, 16040 Havana, Cuba; 2 Department of Biochemistry, Mazankowski Alberta Heart Institute, Faculty of Medicine and Dentistry, University of Alberta, Edmonton, AB T6G 2H7, Canada

**Keywords:** MMP, COVID-19, SARS-CoV-2, pathogen, metabolic tolerance, inflammation, metabolism, AMPK

## Abstract

Many individuals infected with the severe acute respiratory syndrome coronavirus-2 (SARS-CoV-2) develop no or only mild symptoms, but some can go on onto develop a spectrum of pathologies including pneumonia, acute respiratory distress syndrome, respiratory failure, systemic inflammation, and multiorgan failure. Many pathogens, viral and non-viral, can elicit these pathologies, which justifies reconsidering whether the target of therapeutic approaches to fight pathogen infections should be (a) the pathogen itself, (b) the pathologies elicited by the pathogen interaction with the human host, or (c) a combination of both. While little is known about the immunopathology of SARS-CoV-2, it is well-established that the above-mentioned pathologies are associated with hyper-inflammation, tissue damage, and the perturbation of target organ metabolism. Mounting evidence has shown that these processes are regulated by endoproteinases (particularly, matrix metalloproteinases (MMPs)). Here, we review what is known about the roles played by MMPs in the development of COVID-19 and postulate a mechanism by which MMPs could influence energy metabolism in target organs, such as the lung. Finally, we discuss the suitability of MMPs as therapeutic targets to increase the metabolic tolerance of the host to damage inflicted by the pathogen infection, with a focus on SARS-CoV-2.

## 1. Introduction

The emergence of any new pathogen is a perpetual threat to the health and social well-being of humans, as clearly evidenced by the ongoing coronavirus disease-19 (COVID-19) pandemic caused by the severe acute respiratory syndrome coronavirus-2 (SARS-CoV-2). A major challenge for public health systems is the length of time required for the design and mass-production for population-wide access of pathogen-specific and highly efficacious therapeutic approaches (such as vaccines).

Could these challenges be met through the identification of broad-spectrum therapeutic strategies for treating the universe of pathologies commonly caused by pathogens as opposed to neutralizing the pathogens per se?

The predominant approach to fighting pathogen infections is to design effective pathogen-specific vaccines or antimicrobials. These therapeutic approaches can only be designed after the pathogen is known. Making pathogen-specific vaccines or antimicrobials massively accessible takes time and is costly. Resistance mutations can enable the pathogen to evade being neutralized. The design of pathogen-specific therapeutics must be re-started every time a new pathogen emerges [1,2,3].

A less explored complementary therapeutic approach that deserves attention is to enhance host tolerance to disease. Tolerance to disease is a host’s defense strategy aimed at reducing direct tissue damage by pathogens or minimizing immunopathology (e.g., indirect negative impact such as inflammatory damage, changes in host metabolism, excessive extracellular matrix remodeling, and other disruptions of physiological functions that may be caused by the immune response against the pathogens) [1,2,4,5].

The COVID-19 disease severity spectrum ranges from asymptomatic to severe disease. While a significant proportion of individuals who test positive for SARS-CoV-2 develop no symptoms or only mild symptoms (i.e., seem to be relatively tolerant), some become severely ill, showing a hyper-inflammatory response that deregulates the metabolism of target organs with potentially deadly consequences. For instance, among >44,000 adults with COVID-19 in China, 81% showed mild disease, 14% had severe disease, and 5% became critically ill, with an overall case fatality rate of 2.3% and a case fatality rate of 49% among those patients with critical disease [6]. Over 94% of children in China have developed no symptoms, mild disease, or moderate disease, although 5% have developed severe disease and <1% have became critically ill [7,8,9]. Overall, the severity of COVID-19 is magnified by pre-existing comorbidities invariably associated with inflammation and metabolic abnormalities—over 70% of hospitalized cases have one or more pre-existing conditions such as hypertension, heart disease, obesity, diabetes, cancer, and a compromised immune system what occurs in individuals with autoimmune disease (e.g., rheumatoid arthritis) or acquired immunodeficiency (e.g., caused by HIV infection) [10,11]).

Whether or how tolerance mechanisms that act by limiting the damage caused by SARS-CoV-2 burden or immunopathology can be therapeutically manipulated in SARS-CoV-2-infected individuals is unknown.

**Figure 1:**
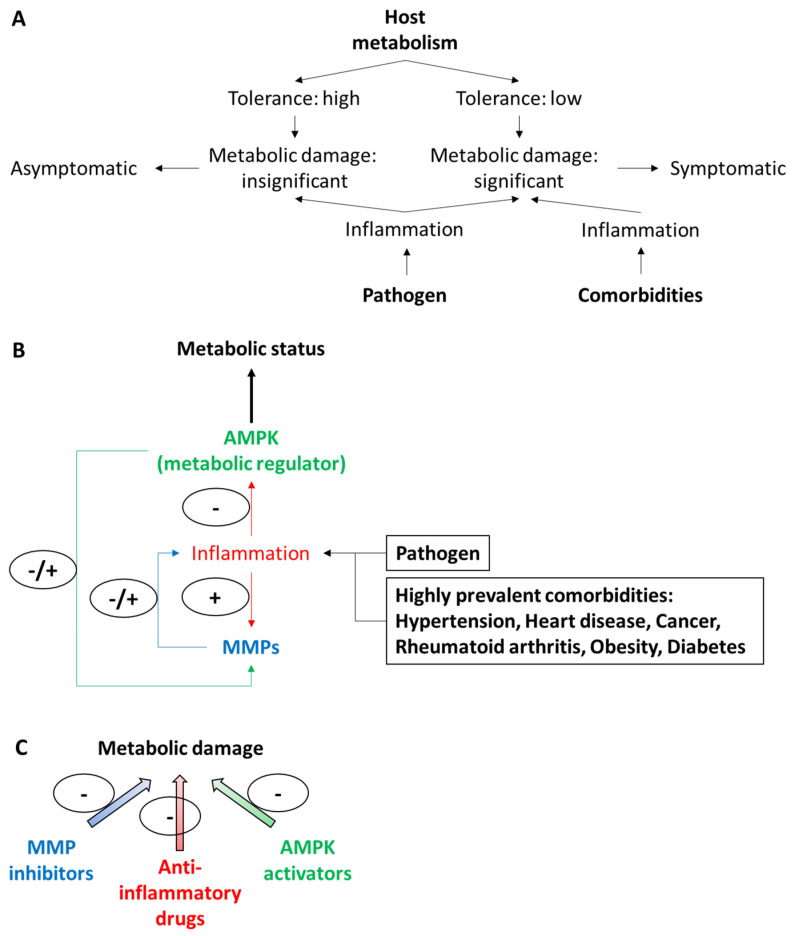
Representation of the proposed hypothesis. (**A**) Individuals can be highly or poorly tolerant to infection with new pathogens including viruses. (**B**) Subjects poorly tolerant to SARS-CoV-2 infection and the effects of COVID-19 tend to exhibit overproduction of proinflammatory cytokines (such as interferons α and γ, interleukins -1β, -6, -12, -18, and -33; transforming growth factor-β; and tumor necrosis factor-α) and chemokines (such as CCL2, 3, and 5 and CXCL8, 9, and 10) [12]. Excessive vascular inflammatory response with the abrupt development of the mentioned cytokine storm can cause severe pulmonary pathologies including acute respiratory distress syndrome, organ damage, and (eventually) a decrease of SARS-CoV-2-infected patients [12]. Increased levels of inflammation-regulated transcription factors (e.g., nuclear factor NF-κB and mature SREBP genes) have been strongly implicated in the development of the cytokine storm in patients (including intensive care unit and deceased subjects) with severe COVID-19 [12]. The activation of SREBP genes will exacerbate cholesterol and fatty acid biosynthesis, which could be a form of innate protection mechanism because cellular cholesterol is thought to participate in the inactivation of SARS-CoV-2 and also in the exocytosis of SARS-CoV-2. Additionally, subjects with severe COVID-19 show (i) altered the pulmonary metabolism and the metabolism of other extra-pulmonary organs and (ii) dysregulated extracellular matrix remodeling in the lung associated with the elevated cell expression, activity, and release of MMPs. SARS-CoV-2 related pulmonary pathologies (such as ALI/ARDS) are exacerbated in subjects with comorbidities associated with inflammation and metabolic anomalies [1,10,11]. (**C**) MMP inhibitors can increase the efficacy of AMPK activators in the setting of COVID-19. Therefore, we suggest that cocktails comprising broad-spectrum metalloproteinase inhibitors at low doses (to avoid excessive toxic effects) and AMPK activators can be used as therapeutic approaches to enhance tolerance against the metabolic damage associated with SARS-CoV-2 in symptomatic patients with COVID-19. Moreover, targeting ubiquitously expressed inflammatory transcription factors (such as NF-κB and SREBP-2) that may be upregulated in severe COVID-19 ([13] and citations therein) can be combined in this approach to hinder the cytokine storm and lung damage in corresponding SARS-CoV-2-infected patients. SARS-CoV-2: severe acute respiratory syndrome coronavirus-2; COVID: coronavirus disease; CCL: CC-chemokine ligand; CXCL: CXC-chemokine ligand; NF-κB: nuclear factor-κB; SREBP: sterol regulatory element binding protein; MMP: matrix metalloproteinase; AMPK: 5′-adenosine monophosphate AMP-activated protein kinase; ALI: acute lung injury; ARDS: acute respiratory distress syndrome.

In this opinion essay, which is part of a Special Issue of Biomolecules entitled “Matrix Metalloproteinases (MMPs) in Health and Disease in the Times of COVID-19”, we recapitulate the implication of MMPs in injurious processes in pulmonary pathologies and bring attention to recent clinical and biochemical evidence suggesting an increase in the levels of MMPs in COVID-19 patients, along with the significant metabolic deregulation of target organs. We also discuss a mechanism by which the excessive activity of MMPs could cause damage to the host by deregulating the metabolism of target organs. We suggest that MMP activity can also influence host tolerance against COVID-19 and, perhaps also, other pathogen-associated diseases (Figure 1).

## 2. MMPs and Their Implications in Pulmonary Pathologies

MMPs are a 23-member family of zinc-dependent and Ca^2+^-containing secreted endoproteinases that cleave a broad spectrum of substrates, including extracellular matrix components (collagens, fibronectin, and elastin), soluble metabolic mediators (e.g., apolipoproteins), secreted and extracellular matrix-anchored growth factors, chemokines, and cytokines [14]. In the lung, metalloproteinases are derived from inflammatory cells (such as neutrophils, alveolar macrophages, and eosinophils) and parenchymal cells (such as pulmonary endothelial cells, type II epithelial cells, and interstitial fibroblasts) [15]. Though MMP expression and activity is very limited in healthy lung tissues, many members of the metalloproteinase family (such as MMP-7, MMP-8, MMP-9, and MMP-12) may be upregulated by diverse factors such as cell-to-extracellular matrix and cell-to-cell interactions, cytokines, and exogenous stimuli or downregulated by cytokines such as interleukin (IL)-4, IL-10, and interferon (IFN)-γ [16]. MMPs are strongly regulated in the lung at the transcriptional, posttranslational, and translational levels of expression by means of a variety of factors such as: (i) transcription factors (e.g., early growth response gene product 1 that induces MMP-14, globin transcription factor 2 that activates the MMP-2 promoter, nuclear factor kappa B, signal transducer and activator of transcription 3 that represses MMP-9 and MMP-12, and activator protein 1 family members that activate the MMP-2 promoter); (ii) growth factors (e.g., epidermal growth factor receptor that up-regulates MMP-9 and MMP-14 expression); (iii) chemokines (e.g., C10/CC-chemokine ligand 6 (CCL6) that increases MMP-2 and MMP-9); (iv) cytokines (e.g., tumor necrosis factor (TNF)-α, interleukins such as IL-1, IL-6, and IL-13 that increase MMP-2 mRNA); (v) endogenous inflammatory intermediates (e.g., reactive oxygen species that activate transcription factors related to MMP gene expression); (vi) environmental factors (e.g., air pollution (which increases MMP-2, MMP-7, and MMP-9) and cigarette smoke (which increases MMP-9 and MMP-12)); (vii) pathogen-derived mediators (e.g., *Mycobacterium tuberculosis,* which up-regulates MMP-1 and stimulates the expression of MMP-9); (viii) mechanical stress (e.g., mechanical ventilation); (ix) the cellular compartmentalization/location of MMPs (e.g., in inflammatory cells); (x) activation state; and (xi) tissue inhibitor of metalloproteinases-1, 2, 3, and 4 (TIMP-1, 2, 3, and 4), which inhibit MMPs in the lung ([15] and citations therein).

MMPs may modulate proper physiological processes (such as the development of the lungs) and responses to exogenous stimuli (e.g., MMP-9-assisted pulmonary epithelial repair) [16]. Additionally, MMPs play key roles in lung immunity by facilitating the influx or egression of inflammatory cells, modulating the activities of chemokines and cytokines, activating defensins (e.g., MMP7-activated antimicrobial peptides), and mediating intercellular signaling (e.g., by processing proteinase activated receptor 1 on the cell membrane surface) [16]. However, the deregulation of the protease–antiprotease balance involving MMPs and their natural inhibitors, the disturbance of the tightly controlled MMP activity, and the improper secretion of several MMPs can have strong detrimental effects. These include the induction of inflammatory responses (e.g., in the airways), excessive tissue remodeling, and, ultimately, tissue destruction. Consequently, MMPs are crucial components of the processes leading to the emergence of acute and chronic lung inflammatory conditions.

This notion has been confirmed in studies with MMP-deficient mice that have shown how individual MMPs may be implicated in lung pathologies [17,18,19,20].

In cigarette smoke-induced chronic obstructive pulmonary disease (COPD), *Mmp12^−/−^* mice show reduced emphysema and inflammation reactions in the lung. In ovalbumin- and *Aspergillus fumigatus*-induced asthma, (i) *Mmp9^−/−^* mice show an interrupted migration of eosinophils and neutrophils into the bronchoalveolar lavage fluid and a considerable accumulation of cells with apoptotic phenotype in the lungs; (ii) *Mmp2^−/−^* mice show an interrupted trafficking of eosinophils into the bronchoalveolar lavage fluid, a considerable accumulation of lung cells with an apoptotic phenotype, and augmented predisposition to asphyxiation; (iii) *Mmp9^−/−^*/*Mmp2^−/−^* mice show inflammatory cell egression mainly controlled by MMP-9.

In bleomycin-induced interstitial lung disease, (i) *Mmp8^−/−^* mice show reduced IL-10 processing and less fibrosis, (ii) *Mmp9^−/−^* mice show diminished alveolar bronchiolization, and (iii) *Mmp7^−/−^* mice show a deficient migration of neutrophil cells into the alveolar space, reduced alveolar fibrosis at advanced stages, less lethality, and decreased fibrosis.

In acute lung injury (ALI), (i) *Mmp8^−/−^* mice under high-pressure ventilation show reduced pulmonary edema and permeability, as well as diminished histological damage; (ii) *Mmp8^−/−^* mice under mechanical ventilation show augmented alveolar permeability; (iii) *Mmp8^−/−^* mice treated with bacterial endotoxin (lipopolysaccharide) show an accumulation of polymorphonuclear cells in the alveolar spaces; (iv) *Mmp8^−/−^* mice treated with bleomycin show higher alveolar capillary barrier damage, an accumulation of polymorphonuclear cells in the alveolar spaces, an increased pulmonary elastance, and augmented mortality; (v) *Mmp9^−/−^* mice treated with lipopolysaccharide show the development of emphysema; (vi) *Mmp13^−/−^* mice under hyperoxia show higher inflammation due to a lack of monocyte chemoattractant protein-1 cleavage by MMP-13; and (vii) *Mmp3^−/−^* mice treated with immunoglobulin G show less lung damage.

Overall, the emerging view is that MMPs are implicated in all lung pathologies through their ability to process the extracellular matrix proteins and non-matrix mediators of lung damage (e.g., cytokines, chemokines, and surface receptors) [18]. Moreover, growth factors, cytokines, and chemokines influence the expression and secretion of MMPs [21,22,23,24] in both lung parenchymal and non-parenchymal cells in the settings of pulmonary conditions, e.g., MMP-1, MMP-2, MMP-8, MMP-9, MMP-12, and MMP-14 contribute to COPD-associated pulmonary damage; MMP-1, MMP-2, MMP-3, MMP-7, MMP-8, MMP-9, MMP-12, MMP-14, and MMP-25 are associated with asthma; MMP-1, MMP-2, MMP-3, MMP-7, MMP-8, MMP-9, MMP-11, MMP-12, and MMP-13 contribute to ALI and acute respiratory distress syndrome (ARDS); and MMP-1, MMP-2, MMP-3, MMP-7, MMP-8, and MMP-9 contribute to idiopathic pulmonary fibrosis [15,25,26,27,28,29]. Obviously, no single MMP is a decisive mediator of any specific pulmonary pathology—each MMP plays an individual role at specific periods in the progression of any given pathology, and potential functional redundancy, based on the numerous overlapping substrate molecules that exist at the site of activity for MMPs, must be considered to describe lung pathologies [26]. Details of the roles played by individual and subsets of MMPs implicated in inflammatory and fibrotic lung disorders can be found in Table 1.

In pathogenic SARS-CoV-2 infection leading to ALI/ARDS, deregulated inflammation involves an increase in the levels of proinflammatory cytokines, several other inflammatory mediators (e.g., chemokines), and inflammatory transcription factors (such as nuclear factor kappa B and sterol regulatory element binding proteins (SREBPs)) [13]. Increased inflammatory mediators activate inflammatory cells (such as neutrophils and alveolar macrophages), which liberate proteases, possibly including MMPs and oxygen radicals, thus producing more pulmonary damage (such as increased vascular leakage and enhanced endothelial and epithelial cell apoptosis) and eventually leading to death [13]. We expect future work will find and report more evidence that implicates excess MMP activity in COVID-19.

## 3. COVID-19 Clinical Characteristics

### 3.1. COVID-19 Severity Spectrum May Be Related to Metabolic Changes in the Lung and Extra-Pulmonary Organs

Coronaviruses are RNA viruses that hijack the host’s translational machinery to generate viral proteins [38]. Seven coronaviruses are known to infect humans, and four of them are endemic human coronaviruses that cause common colds annually. At least three zoonotic coronaviruses have caused outbreaks in humans: severe acute respiratory syndrome coronavirus-1 (SARS-CoV, which had an outbreak in 2002–2003), Middle East respiratory syndrome-coronavirus (MERS-CoV, which had an outbreak in 2012), and SARS-CoV-2 (which is responsible for the current COVID-19 pandemic) [38,39]. SARS-CoV, MERS-CoV, and SARS-CoV-2 infect the human host by binding to cell surface receptors, e.g., SARS-CoV, MERS-CoV, and SARS-CoV-2 infect the human host by binding to cell surface receptors such as angiotensin-converting enzyme 2 (ACE2), CD147, and CD26, respectively, via the trimeric spike protein and the co-receptor (TMPRSS2). Spike comprises the S1 and S2 subunits, which share an extracellular domain, while the S1 subunit comprises the N-terminal domain, whose function is unclear, and the receptor binding domain (RBD) through which these coronaviruses attach to ACE2 during infection [39].

Many vaccine candidates aim to induce the production of host-neutralizing antibodies targeting the RBD [40,41]. A drawback of this approach is that the resistance-inducing mutations on the RBD could invalidate the neutralization of the virus by RBD-targeting antibodies [42,43]. Alternative antibody-based therapeutic strategies aiming at non-RBD regions such as the N-terminal domain [44] or non-antibody drug cocktails remain untested.

Recently, the idea of complementary approaches has gained ground, as biochemical pathways in the host could be rerouted to enhance the host’s tolerance to pathogen burden and immunopathology, i.e., to enhance an innate defense mechanism well-known to enable humans, animals, and plants to defend themselves from many types of pathogens, including viruses, bacteria, and parasites, through adaptations or mutations that effectively repair the damage to the host induced by either the pathogen or the host immune response [1,2,4,5]. However, the clinical history of the patient could limit the effectiveness of the innate tolerance mechanisms and the therapeutic approaches designed to enhance tolerance.

One of the perplexing features of SARS-CoV-2 infection is the broad spectrum of disease severity that ensues. Approximately 25–50% of SARS-CoV-2-infected individuals report no symptoms. These asymptomatic individuals maintain their health over time. Approximately 80% of infected people, who go on to develop symptoms, develop only a ‘mild’ disease, but the other 20% develop severe COVID-19, which involves pneumonia, ARDS and respiratory failure, septic shock, and multiorgan failure that affects the functional integrity of extra-pulmonary systems (e.g., oxygen transport, coagulation and cardiac, brain, and renal functions).

The phenotype of these severe and critical COVID-19 cases is unlikely to be driven by the SARS-CoV-2 infection per se. Many of its features are observed in response to many other pathogens including the SARS and MERS coronaviruses, influenza virus, and *Mycobacterium tuberculosis*. Furthermore, the host hyperbolic pro-inflammatory response to the SARS-CoV-2 infection is likely to trigger multisystem dysfunction, the damaging consequences of which are not effectively contained or repaired by the host’s metabolic response. Increasing evidence is connecting the host’s metabolic response in the pulmonary and extra-pulmonary organs in patients with severe COVID-19 with a deficiency of activity of AMP-activated protein kinase (AMPK), a major sensor of energy metabolism in all cells.

### 3.2. Metabolic Alterations in the Lungs

Lung disease is a hallmark of severe COVID-19 related to the pathological disruption of the alveolar (epithelial)–capillary (endothelial) barrier in response to SARS-CoV-2 infection. In healthy individuals, the alveolar–capillary barrier normally serves to regulate fluid exchange across the vasculature and lung epithelium, thus protecting one from pulmonary edema, congestion, and respiratory failure, all of which can develop in response to pathogen infection [45]. Critically ill COVID-19 patients typically have pneumonia, and ARDS is one the major causes of death in these patients [46]. ARDS develops when the alveolar–capillary barrier integrity is perturbed in a way that exacerbates vascular permeability to fluid and the extravasation of leucocytes [47]. Patients also present with inflammation in alveolar capillary walls (vasculitis) [48] and massive neutrophil infiltration in pulmonary capillaries, acute capillaritis, and the extravasation of neutrophils into the alveolar space [49,50]. As the pulmonary damage occurs, there is an irreversible decline in lung function due to pulmonary fibrosis [51] (i.e., extracellular matrix deposition around alveoli resulting from uncontrolled extracellular matrix metabolism), which disrupts gas exchange and accelerates respiratory failure. There is no cure for fibrosis per se, but it can be curbed by interventions that ameliorate extracellular matrix biosynthesis in response to cytokines or the pharmacological modulation of the activity of MMPs, proteases that govern the fibrosis process through the cleavage and activation/deactivation/processing of inflammatory cytokines, growth factors, and extracellular matrix proteins.

One of the metabolic mechanisms that enables normal endothelial cell function and the maintenance of endothelial barrier integrity is the AMPK pathway in the lungs. AMPK is a serine/threonine protein kinase complex consisting of a catalytic α-subunit (α1 and α2), a scaffolding β-subunit (β1 and β2), and a regulatory γ-subunit (γ1, γ2, and γ3) [52,53,54]. The α1 and α2 subunits exhibit cell-type-specific expression in the myocardium, with AMPK-α1 being expressed by endothelial cells [55] and the α2 catalytic subunit predominantly being expressed in myocytes (cardiac and skeletal) and the liver [55,56,57,58]. AMPK phosphorylates both metabolic enzymes (e.g., acetyl-coenzyme A CoA carboxylase (ACC), 3-hydroxy-3-methyl-glutaryl HMG-CoA reductase) and SREBPs, which are transcription factors for ACC and HMG-CoA reductase [53,54]. Activated by oxidative and metabolic stress including nutrient deprivation, AMPK activity stimulates the catabolism and inhibits the biosynthesis of fatty acids, glucose, and protein [53,54,59].

AMPK activity can inhibit cell growth through the phosphorylation of mediators in various pathways [59,60,61,62]: (a) the calcineurin/nuclear factor of activated T cells pathway, (b) the mitogen-activated protein kinase pathway, (c) the mammalian target of rapamycin (mTOR)/p70 ribosomal S6 protein kinase pathway, (d) the eukaryotic elongation factor-2 pathway, and (e) the O-linked β-N-acetylglucosamine acylation O-GlcNAcylation pathway. AMPK activity deficiency can be ameliorated by drugs that indirectly increase the intracellular levels of AMP (biguanides like metformin, thiazolidinediones like troglitazone, and polyphenols like resveratrol) and AMP mimetic drugs that directly bind to and allosterically activate AMPK such as A-769662 [53,63].

Lung AMPK activity deficiency has been observed in acute lung-injury models, such as mice exposed to pathological doses of bacterial endotoxins [64]. Mice lacking catalytically active AMPK (AMPKα-knockout mice) are predisposed to endothelial barrier dysfunction, inflammation, edema, and congestion [65]. Pharmacological AMPK activation improves endothelial barrier integrity and increases tolerance against acute lung injury in these AMPK activity-deficient mice [65,66].

### 3.3. Metabolic Alterations in Extra-Pulmonary Organs

In addition to the lungs, COVID-19 patients present with damage to extra-pulmonary systems including the heart, skeletal muscles, brain, kidney, and gastrointestinal organs. While the causes of these extra-pulmonary events are not clear, these tissues express ACE-2. This means that the damage of these organs can result from SARS-CoV-2 infecting these tissues, as well as being consequence of the host hyper-inflammatory response to the infection. However, other mechanisms likely contribute to extra-pulmonary metabolic alterations. Respiratory failure causes hypoxia, and the limited delivery of oxygenated blood can acutely affect kidney function, thus leading to the accumulation of toxic levels of metabolites in the blood [67,68]. Some COVID-19 patients experience heart damage and abnormal blood clotting during the infection [69,70]. Impaired cardiac function can reduce the supply of oxygenated blood to extra-pulmonary organs, thus contributing to systemic hypoxia and non-cardiac organ failure. The heart obtains energy from fatty acid oxidation under normal physiological conditions and from glucose oxidation in disease conditions. Fatty acid and glucose oxidation are both governed by myocardial AMPK activity. Whether AMPK is a candidate therapeutic target in the heart and lung in the settings of COVID-19 is unknown. Patients with hypertensive heart disease and ischemic injury exhibit an increased activity of MMPs, exacerbated extracellular matrix synthesis, and cardiomyocytes hypertrophy, which are associated with an upregulation of glycolysis in cardiomyocytes [71]. These features indicate a potential mechanistic connection between extracellular matrix metabolism, which is controlled by MMPs, and the switch from fatty acid oxidation to glycolysis—two metabolic processes controlled by AMPK. Ultimately, the switch from fatty acid oxidation to glucose utilization ensures enough myocardial contractile efficiency amid low oxygen tensions (i.e., optimal ATP production despite limited oxygen consumption). Increasing glucose oxidation and decreasing fatty acid oxidation to improve cardiac efficiency [72] could be beneficial in COVID-19 patients with cardiac disease.

## 4. MMP-Involvement in Organ Damage Warrants Investigation

### Role of MMPs, Inflammatory Response, and AMPK

A controlled inflammatory response serves to kill invading pathogens, but an exaggerated response with high levels of many cytokines is harmful for the host [73,74,75]. MMPs are major regulators of tissue and organ homeostasis. However, MMPs are mediators of tissue and organ damage when their activity is pathologically elevated, as demonstrated by earlier research for MMPs in comorbidities that accompany severe COVID-19 cases [1,10,11]. In response to infection with SARS-CoV-2 (and possibly other pathogens), the host’s immune and target cells are likely to release MMPs because one of the physiological stimuli that cause all cells to alter their production and release MMPs is inflammation, which itself is modulated by the MMP-mediated cleavage of cytokines and chemokines [25,26,29,76,77,78,79]. SARS-CoV-2 infection and the comorbidities that accompany COVID-19 are pro-inflammatory on their own, and their superimposition exacerbates inflammation.

MMP activity normally governs the metabolism of extracellular matrix proteins, as well as substrates that are normally anchored either at the extracellular matrix or cell membrane such as cytokines, growth factors, and cell membrane receptors. The uncontrolled MMP-processing of extracellular matrix components and cytokines can be highly pro-inflammatory on its own and affects the physiology of the lung and other organs [25,26,29,76,80,81].

Pro-inflammatory cytokines and chemokines influence the expression of MMPs [26,29,81,82]. Conversely, MMPs can process a spectrum of cytokines and chemokines catalyzing their activation and deactivation [23,76]. Many active cytokines and chemokines stimulate the production of pro-TNF-α in target cells. The proteolytic conversion of the inactive (latent) form pro-TNF-α results in active and highly pro-inflammatory TNF-α. One of the mechanisms by which inflammatory signaling deregulates the metabolism of target cells is the deactivation of AMPK. The deactivation of AMPK by TNF-α in skeletal muscle and adipose tissue in mice with obesity and diabetes involves the de novo synthesis of protein phosphatase 2C PP2C, a ubiquitous Ser/Thr protein phosphatase [83,84,85] that dephosphorylates p-AMPK-α (Thr172), effectively deactivating AMPK. Since PP2C is ubiquitously expressed [86,87], TNF-α likely inhibits AMPK through the PP2C mechanism in many organs. We suggest that alterations in MMP proteolytic activity leading to increased TNF-α levels could thus result in the deactivation of AMPK through the PP2C mechanism in the lung and extra-pulmonary organs, leading to the deregulation of many downstream enzymes required for the catabolism of fatty acids, proteins, and glucose for energy, as well as enzymes required for the synthesis of these substrates.

There is evidence that AMPK activation can suppress MMP-2, -9, -13, and -14 expression in adipose tissue [88] and embryonic fibroblasts [89] while increasing the cell surface expression of MMP-14 and the activation of MMP-2 in cardiac fibroblasts [90]. Conversely, the proteolytic cleavage of cytokines and chemokines by MMPs is likely to impact the AMPK pathway. Thus, there is a cross-talk between the MMP and AMPK pathways that could influence the metabolism of target cells.

Cytokines, chemokines, and MMP levels may define an immune-based biomarker system of COVID-19. A multivariable analysis of patients’ first samples associated an increase of CCL2, IL-15, secreted tumor necrosis factor receptor superfamily member 1A sTNFRSF1A, IL-6, MMP-9, IL-2, or IL-10 with COVID-19 mortality [91]. A hyper-inflammatory response, which presents as a cytokine storm, exacerbates COVID-19 severity with high levels of serum IL-6, IL-8, TNF-α, and IL-1β, thus indicating low chances of patient survival [92,93]. When adjusting for disease severity, inflammation markers, hypoxia, demographics, and pre-existing comorbidities such as type 2 diabetes or hypertension, high levels of serum IL-6 and TNF-α independently predict death [92]. The utilization of IL-6 receptor inhibitors, such as tocilizumab, to treat patients with COVID-19 has yielded mixed clinical results including failure—perhaps because monocytokine therapies tend to fail in other complex immunological conditions like sepsis and multi-organ failure [94]. It is unrealistic to expect that a single cytokine drives all the inflammatory and metabolic damage initiated by the SARS-CoV-2 infection and further complicated by comorbidities. This may be why, in contrast to monocytokine therapies, more global immune-modulating treatments, such as dexamethasone or multi-cytokine-directed cocktails, are proving more effective in treating COVID-19 [94].

Little is known about the cellular origin and mechanisms implicated in the production and release of MMPs in the settings of SARS-CoV-2 infection [30]. Studies on MMP-9 may shed some light into the specific roles played by MMPs in severe COVID-19 pathogenesis. MMP-9 is a 92 kDa gelatinase that participates in ALI/ARDS in patients with asthma, pulmonary fibrosis, and chronic obstructive pulmonary disease [18,30,95]. There is a distinct and early increase in circulating MMP-9 in COVID-19 patients with respiratory failure. This increase likely involves the coronavirus-induced MMP-9 release from neutrophils, which promote the inflammation and degradation of the alveolar capillary barrier [18]. The emerging data suggest that MMP-9 may be an early indicator of and candidate therapeutic target in respiratory failure and lung fibrosis in COVID-19 patients. Recent data have linked MMP-9 levels to vitamin D in respiratory infections and COVID-19. Vitamin D deficiency has been observed in many patients with severe COVID-19. In a study of 171 healthy British Bangladeshi adults, vitamin D levels were inversely correlated with circulating MMP-9 levels [96], while a vitamin D metabolite (1α,25(OH)_2_D_3_) was found to inhibit MMP-9 gene expression, secretion, and activity in the setting of various respiratory infections [97]. These data suggest possible links between MMP-9 and lipid metabolism in COVID-19, which is not surprisingly based on previous (COVID-19-unrelated) studies by many investigators including our group (for MMP-2, MMP-7, and MMP-9, as well as their tissue inhibitors TIMP-2 and TIMP-3) [14,78,98,99,100,101,102,103].

## 5. Improving Metabolism Through Manipulation of the Possibly Synergic or Cooperative Actions of MMPs, Cytokines, and AMPK Pathways on Systemic Metabolism

Given that MMPs can promote the activation of TNF-α and TNF-α signaling promotes the deactivation of AMPK, we suggest that the convergence of increased MMP proteolytic activity, increased TNF-α levels, and reduced AMPK activity could have a synergic and negative impact on the metabolic pathways regulated by AMPK in COVID-19 patients (Figure 1B). If so, the cross-talk among MMPs, TNF-α, and AMPK is a candidate therapeutic target to mitigate the metabolic damage associated with COVID-19 (Figure 1B,C).

Cooperative effects including addition and synergism are efficient ways for amplifying the effects induced by low levels of stimuli, e.g., that toxicity is reduced when effective drug concentrations are lowered sufficiently. Could MMP inhibitors at doses that reduce TNF-α activation synergistically increase the efficacy of AMPK activators? If so, then cocktails comprising low doses (to minimize toxicity) of inhibitors of TNF-α-activating metalloproteinases and AMPK activators could be designed to normalize AMPK activity.

Currently, there are no Food and Drug Administration-approved MMP inhibitors for treating cardiovascular disorders except for doxycycline, which is approved as an MMP inhibitor by Health Canada and the U.S. FDA for the treatment of periodontitis and rosacea. Early trials with various other inhibitor structures showed toxic effects at high doses in patients with left ventricular (LV) remodeling after myocardial infarction [104,105] and cancer [106]. However, recent clinical trials have explored whether decreasing the proteolytic activity of MMPs [105,107] or increasing the kinase activity of AMPK [108,109] is effective in treating cardiovascular conditions. Encouraging results have been reported for doxycycline (MMP inhibitor) in reducing adverse LV remodeling in ST-segment elevation myocardial infarction (STEMI) patients (Tetracycline (Doxycycline) and Post Myocardial Infarction Remodeling TIPTOP trial) [105] and for metformin (AMPK activator) in reducing left ventricular mass indexed to height in non-diabetic patients with coronary artery disease (MET-REMODEL trial) [108]. These results add to overwhelming evidence implicating MMPs and AMPK in the pathogenesis of adverse cardiac remodeling in hypertension, myocardial infarction, and heart failure [59,61,110,111,112,113,114,115,116,117,118,119,120] (all of which are comorbidities in COVID-19).

Could cocktails comprising MMP inhibitors at low doses (to avoid excessive toxic effects) increase the efficacy of AMPK activators in the setting of COVID-19? Support for the potential use of MMP inhibitors at low doses lies in the encouraging results of recent clinical trials using low doses of doxycycline to inhibit MMPs. Doxycycline is an antibiotic with broad-spectrum MMP inhibitory activity (IC_50_ (for gelatinases) = 50 µM) [121,122]. In the TIPTOP trial [105], low-dose doxycycline (100 mg b.i.d. (approximately 3 mg/kg/day considering that the participants’ average weight was 70 kg) for seven days) reduced LV end-diastolic volumes index in patients with acute STEMI and LV dysfunction. Based on these promising results, two new trials were approved in Australia (ACTRN12618000467235) and Canada (ClinicalTrials.gov Identifier: NCT03508232). In another trial (MET-REMODEL) conducted for 12 months, metformin (2000 mg daily dose (approximately 30 mg/kg/day considering that the participants’ average weight was 70 kg)) improved the LV mass index in patients without diabetes [108]. In mice, a high dose of metformin (200 mg/kg/day) seems necessary to prevent agonist-induced LV hypertrophy [61,123]. Why a high dose of metformin is required to activate AMPK is unknown [52]. However, we hypothesize that the production of inflammatory molecules (such as TNF-α) associated with adverse cardiac remodeling could reduce AMPK activity, thus mitigating the potency of AMPK activators such as metformin. Likewise, the attenuation of the efficacy of AMPK activators may occur during the innate inflammation response of the host to SARS-CoV-2. We think this is an important area that warrants further research, with the possible outcome that the therapeutic actions of MMPs and AMPK activity modulatory drugs could be maximized for use in the treatment of many disorders.

While combining metformin with a broad-spectrum anti-inflammatory treatment (or TNF-α inhibitors) is reasonable, one advantage in choosing an MMP inhibitor as a ‘broad-spectrum anti-inflammatory drug’ at very low doses is that one could obtain a therapeutic cocktail that could (i) have a lower risk of developing unacceptable toxicity, especially if there is a need of long-term use; (ii) dampen inflammation; (iii) block the MMP-dependent excessive cleavage of extracellular matrix components; and (iv) normalize AMPK activity by preventing the deactivation of AMPK by cytokines such as TNF-α with positive end-effects such as mitigating adverse extracellular matrix remodeling or restoring target organ metabolism to limit organ damage by SARS-CoV-2. Obviously, the off-target effects of global MMP inhibition—e.g., on cellular pathways and inflammatory mediators associated with pulmonary damage—warrants investigation [124].

## 6. Limitations

Apart from doxycycline, which exhibits MMP inhibitory actions at sub-antimicrobial concentrations and is approved to treat periodontitis and rosacea, there are currently no Food and Drug Administration-approved MMP inhibitors for treating cardiovascular and pulmonary disorders. It is hoped that the hypothetical approach described in this essay will stimulate the future design of efficacious therapeutic approaches to increase tolerance to the universe of pathologies elicited by SARS-CoV-2 (as well as other pathogens).

## 7. Conclusions

There persists a worldwide need for improved therapeutic strategies that are broadly applicable to mitigate and repair pathogen-induced damage, particularly in COVID-19 patients. Unbalanced levels of a variety of MMPs have been documented in lung disorders such as COPD, asthma, pulmonary fibrosis, and ALI/ARDS, as well as in the inflammatory process related to COVID-19. The modulation of airway remodeling to minimize or avoid pulmonary injury caused by unregulated proteolysis remains poorly understood. However, the disproportionate airway remodeling as a consequence of the rupture in the balance between physiological synthesis and matrix degradation suggests a medicinal role for approaches to modulate MMP activity. In addition to modulating tissue remodeling, MMPs are emerging as important modulators of inflammation and energy metabolism. Inflammation influences both MMP activity and metabolism. Conversely, the MMP cleavage of cytokines, chemokines, and extracellular matrix influences the inflammatory status and, thereby, energy metabolism of target organs—as addressed earlier in this review. One avenue that deserves further research is the possibly synergic or cooperative actions of MMPs, cytokines such as TNF-α, and AMPK pathways that govern cellular metabolism. We suggest that it should be possible to identify new cocktails comprising low doses of metalloproteinase inhibitors and AMPK activators that are more efficacious at improving the metabolism of the host than using metalloproteinase inhibitors or AMPK activators alone. It is hoped that such an approach will increase tolerance to (and be applicable to treat) the universe of pathologies elicited by many pathogens, not just SARS-CoV-2.

## Figures and Tables

**Table 1:** Dysregulation of MMPs in inflammatory and fibrotic lung disorders ([15,16,17,18,26,30,31,32,33,34,35,36,37] and citations therein).

MMPs	Disease	Suggested MMP Role in Disease
MMP-1	Emphysema, which is a chronic obstructive pulmonary disease that is characterized by extensive tissue remodeling, the destruction of small bronchi and alveolar septa, diminished lung plasticity, and ultimately impaired pulmonary oxygenation. Increased MMP-1 levels have been detected in type II pneumocyte in the lung parenchyma of patients with emphysema, as well as in alveolar lining cells and fibroblasts exposed to smoke. Asthma, which is a condition associated with lung inflammation, episodic dyspnea, and airway hyperresponsiveness. Increased MMP-1 levels have been detected in airway smooth muscle cells from asthma and emphysema patients.	Drives pulmonary tissue destruction such as the spontaneous development of air space expansion and emphysema-like changes including the coalescence of the alveolar spaces and disruption of the alveolar walls, as shown in transgenic animal models. Induces airway obstruction and ASMC hyperplasia in asthma by activating the IGF axis. Contributes to altered contractile response of smooth muscle cells induced by Th2 cytokines (IL-4 and IL-13), chiefly in allergic asthma.
MMP-2	Asthma; increased MMP-2 levels have been detected in sputum of patients with this condition. Idiopathic pulmonary fibrosis (IPF), which is a chronic condition (usually lethal due to respiratory failure), characterized by disproportionate extracellular matrix degradation, inflammation, and fibrosis of the lung parenchyma. Pneumonia; increased MMP-2 levels have been detected in an elevated proportion of patients with this condition.	Participates in tissue degradation in emphysema. Aggravates pulmonary hypertension, which may lead to right heart failure and death. Involved in collagen deposition in IPF.
MMP-3	Asthma; increased MMP-3 levels have been detected in bronchial lavages of individuals with this condition.	Involved in airway hyperreactivity and diminished lung function in asthmatic patients, which may be related to the activation of MMP-9 and the augmented synthesis of procollagen I.
MMP-7	Asthma; increased MMP-7 levels have been detected in airway epithelial cells and in lung epithelial cells under the effect of osteopontin. Cystic fibrosis (CF), which is a genetic disorder characterized by malfunctioning of the chloride channels of the airway epithelia and other epithelial linings. Increased MMP-7 levels have been detected in type II pneumocytes in patients with CF	Contributes to airway epithelial damage and inflammation in subjects with severe asthma by cleaving and releasing soluble FasL. Plays a profibrotic effect in IPF.
MMP-8	Exacerbated chronic obstructive pulmonary disease (COPD); COPD is a progressive pathology that has cigarette smoking as the main triggering factor, and is characterized by airway abnormal inflammation, airway flow obstruction, and pulmonary failure. Emphysema; increased MMP-8 levels have been detected in the bronchoalveolar lavage (BAL) fluid of smokers with this condition. Asthma; increased MMP-8 levels have been detected in the BAL fluid of patients with this condition. Pediatric severe chronic lung disease; increased MMP-8 levels have been detected in BAL fluids from preterm babies (before 28 weeks of gestation) who later developed this disease.	Increases collagenolytic activity, impairing the collagen network of the lung and thus contributing to emphysema in adults. Associated with severe granulocytic inflammation in airways caused by allergen exposure in MMP-8-deficient mice. Contributes to the lung harm that happens in the initial phases of chronic lung disease. Linked to bronchiectasis. Involved in severe chronic lung illness in preterm babies where the alveolarization of the saccules alveolaris is starting. Involved in pulmonary fibrosis. Triggers an inflammatory response in the lungs associated with injurious ventilation.
MMP-9	COPD; increased MMP-9 levels have been detected in cells (e.g., alveolar macrophages) and sputum of patients with COPD (e.g., in bronchial epithelium and submucosal areas). Emphysema; increased MMP-9 levels have been detected in (i) the lung parenchyma of patients with this condition, (ii) alveolar macrophages from smokers, and (iii) cultured airway macrophages from smokers (at baseline and in response to IL-1b and LPS). Asthma; increased MMP-9 levels have been detected in (i) the sputum of subjects with allergic asthma after flour inhalation; (ii) the BAL fluid (mainly associated with airway neutrophils and less with alveolar macrophages—and maybe with eosinophils), sputum, serum, and bronchial tissues of asthmatic patients who have chronic submucosal inflammation; and (iii) samples from workers exposed to toluene diisocyanate-occupational asthma. Rapidly progressive IPF and IPF-usual interstitial pneumonia (UIP); increased MMP-9 levels have been detected in BAL fluid and pulmonary tissues in patients with these conditions. Acute lung injury (ALI), which is a disease characterized by the disruption of the diffuse alveolar–capillary wall, the invasion of circulating inflammatory cells, and deficient oxygenation caused by respiratory syncytial virus (RSV) infection and other viral infections. Increased MMP-9 levels have been detected in respiratory secretions of children with ALI. Acute respiratory distress syndrome (ARDS), which is the most severe manifestation of ALI and is characterized by excessive systemic inflammation, an augmented permeability of the alveolar epithelial–endothelial capillary barrier, tissue damage, and acute respiratory failure. Increased MMP-9 levels have been detected in (i) the plasma of patients with ARDS, (ii) BAL fluid corresponding to the acute phase of ARDS, and (iii) the BAL fluid of patients with ARDS associated with severe trauma or septic shock. Mechanically ventilated infants with RSV-induced respiratory failure. Infection with coronavirus; increased MMP-9 levels have been detected in the circulation of COVID-19 patients with respiratory failure.	Implicated in a variety of illnesses, including COPD, emphysema, asthma (e.g., by enabling cell migration and airway inflammatory reaction, increased susceptibility to airway remodeling upon exposure to cigarette smoke resulting in thickening of airway walls, and bronchopulmonary dysplasia), ALI, CF, neutrophilic inflammation-mediated VILI (which is a severe lung injury associated with high-pressure mechanical ventilation), mechanically ventilated infants with RSV-infected lungs, and interstitial lung diseases (such as IPF-UIP and diffuse alveolar damage). Promotes eosinophil migration into the airways from asthmatic patients. Stimulates the degradation of the alveolar capillary barrier and inflammation, further promoting the migration of inflammatory cells (e.g., neutrophil recruitment through the generation of collagen fragments with chemotactic properties) and the damage of lung tissue. Associated with diffuse alveolar injury in hyperoxia-induced ALI in pigs. Linked to inflammation-induced tissue remodeling. Associated with markers of basement membrane disruption, in the acute phase of ARDS. Exacerbates lung tissue remodeling in rat lungs with hyperoxia. Implicated, when MMP-9/TIMP-1 ratio is less than 1, in the evolution of ARDS to fibroproliferation. Contributes to the disruption of alveolar epithelial basement membrane and increases fibroblast migration to alveolar spaces in the initial stage of lung fibrosis. Associated with respiratory failure in COVID-19 patients.
MMP-12	COPD; increased MMP-12 levels have been detected in the BAL fluids, bronchial biopsy tissue, BAL cells, and sputum of patients with COPD. Emphysema; increased MMP-12 levels have been detected in dendritic and bronchial epithelial cells in human lung in response to cigarette smoke.	Participates in the pathogenesis of acute and chronic respiratory disorders (such as COPD, cigarette smoke-induced emphysema, and asthma) when they are induced in ASMC by inflammatory cytokines under the regulation of different mechanisms (such as PI3-K, JNK, ERK, and AP-1 signaling pathways). Partially mediates the IL-13-induced expression of MMP-2, MMP-9, MMP-13, and MMP-14, which results in the development of COPD-related features (such as the accumulation of macrophages, eosinophils, and neutrophils, thus leading to lung inflammation, alveolar enlargement, and increased lung volume). Involved in smoke-induced inflammation and emphysema (e.g., by causing endothelial activation mediated by the release of TNF-α from macrophages, degrading connective tissue, and facilitating neutrophil influx). Facilitates airway inflammation by promoting the migration of inflammatory cells (e.g., macrophages and monocytes) to inflammatory zones. Implicated in airway remodeling by degrading extracellular matrix proteins (e.g., fibronectin, elastin, laminin, type IV collagen, and gelatin) or mediating inflammatory cytokines to stimulate production of other MMPs (MMP-2, MMP-9, MMP-13, and MMP-14) in the lung. In animal models, it facilitates the development of inflammatory processes involving the recruitment of inflammatory cells, increases in cytokine and chemokine levels, and elevation of MMPs (e.g., MMP-2 and MMP-9) levels in lung samples and BAL fluids.
MMP-14	COPD; increased MMP-14 levels have been detected in samples (e.g., BAL fluid from patients with emphysema) from COPD patients upon acrolein induction.	Implicated in COPD pathogenesis.
MMPsubsets	COPD; levels are elevated for (i) MMP-1 and MMP-2 derived from epithelial cells and macrophages, MMP-8 and MMP-9 derived from neutrophils, MMP-12, and MMP-14—all in the pulmonary tissues of COPD patients; (ii) MMP-1, MMP-2, MMP-8, and MMP-9 in the BAL fluid of patients with COPD; and (iii) MMP-2 and MMP-9 in the sputum of patients with COPD. Emphysema; levels are elevated for (i) MMP-1 and MMP-9 in the BAL fluid of patients with emphysema; (ii) MMP-8 and MMP-9 in alveolar macrophages from patients with emphysema; and (iii) MMP-9 and MMP-12 induced by cigarette smoke in human lung. Asthma; levels are elevated for (i) MMP-1, MMP-2, MMP-3, MMP-8, and MMP-9 in the sputum and BAL of asthma patients; (ii) MMP-2, MMP-3, and MMP-14 (and also TIMP-1 and TIMP-2) in the ASMC of patients with bronchial asthma; and (iii) MMP-2 and MMP-12 in the bronchial epithelial, smooth muscle and submucosal glandular cells, BAL fluid, and sputum of subjects with bronchiectasis and asthma. IPF; levels are elevated for (i) MMP-3, MMP-7, MMP-8, and MMP-9 in the lavage fluid of patients with IPF. ALI; levels are elevated for (i) MMP-2, MMP-3, MMP-8, MMP-9, MMP-11, and MMP-12, generated mainly by neutrophil and macrophage cells in the lung secretions of pediatric patients with ALI. ARDS; levels are elevated for (i) MMP-1, MMP-2, MMP-3, MMP-7, MMP-8, MMP-9, MMP-12, and MMP-13 in patients with ARDS; (ii) MMP-2 and MMP-9 in the lung of newborns with ARDS; and (iii) MMP-8 and MMP-9, and to a limited extent for MMP-3, MMP-11, and MMP-12 in the lung secretions of children with ALI/ARDS. Pneumonia; levels are elevated for (i) MMP-8 and MMP-9 in the BAL fluid of patients with pneumonia acquired in hospitals. Ageing lungs; levels are elevated for (i) MMP-9 and MMP-12 (and also TIMP-1 and TIMP-2) in ageing lungs. By contrast, decreased MMP levels are reported for MMP-1 and MMP-2 in ageing lungs. Pulmonary fibrosis; levels are elevated for (i) MMP-2 and MMP-9 in BAL fluid and pulmonary tissue associated with pulmonary fibrosis; (ii) MMP-1 (largely found in bronchiolar and alveolar epithelial cells), MMP-2, MMP-7 (in the epithelium of lungs with IPF and fibrotic lung extracts), and MMP-9 in the lungs of patients with IPF; (iii) MMP-3 and MMP-12 in human alveolar macrophages under the effect of surfactant protein D; and (iv) MMP-2, MMP-8, and MMP-9 in the BAL fluid of patients with CF. Bronchiolitis obliterans, which is an irreversible condition characterized by obstructive airway remodeling. Levels are elevated for (i) MMP-8 and MMP-9 from neutrophils recruited into the lung (and also for TIMP-1) in patients with advanced bronchiolitis obliterans.	MMP-2 and MMP-9: Involved in the pathogenesis (e.g., excessive tissue remodeling) of COPD. MMP-1, MMP-8, and MMP-9: associated with the pathogenesis of emphysema. Predominantly MMP-9 and several other MMPs (such as MMP-1, MMP-2, MMP-7, MMP-8, MMP-12, and MMP-25): associated with asthma pathology. MMP-3, MMP-7, MMP-8, and MMP-9: implicated in pulmonary fibrosis, by facilitating the release of extracellular matrix-derived VEGF, resulting in abnormal capillary permeability and neoangiogenesis. MMP-1, MMP-2, MMP-7, and MMP-9 (and also TIMP-1 and TIMP-2): show an active role in IPF. MMP-8 and MMP-9: associated with the grade of systemic inflammation in patients with ventilator-associated pneumonia. MMP-9 and MMP-12 (and also TIMP-1 and TIMP-2): associated with deteriorated lung function during ageing. MMP-3 and MMP-9: associated with pathogenic roles (e.g., interstitial matrix remodeling, rupture of the basement membranes) in ALI. MMP-2, MMP-9, and MMP-12: mediate structural lung damage (alveolar type I cell injury, pulmonary compliance variations, respiratory failure, and death) in mouse models (e.g., hyperoxia-induced lung disease). MMP-1, MMP-3, and MMP-12: involved in lung innate immune system; pulmonary surfactant proteins induces the expression of these enzymes in human alveolar macrophages. Activated macrophages in the lung clear offending pathogens (viruses, bacteria, and fungi) that enter the airway. MMP-9 and MMP-12: implicated in the effects of IL-1β in lungs of asthmatic patients; in the lungs of adult mice, IL-1β contributes to pulmonary inflammation (e.g., macrophage and neutrophil infiltration) and fibrosis in the airway. MMP-2, MMP-8, MMP-9 (and also TIMP-1) and sometimes MMP-1 and MMP-3: involved in neutrophil-mediated inflammation, extensive tissue remodeling, and the loss of the intercellular junctions and alveolar–capillary barrier that occur in ARDS. These roles are also valid for MMP-3, MMP-7, and MMP-12 in animal models. MMP-1 and/or MMP-3, together with MMP-2, MMP-8, and MMP-9 (and also TIMP-1): associated with more severe disease progression (i.e., higher indices of disease harshness, widespread multiorgan failure, and more mortality) in patients with ALI/ARDS. MMP-2 and MMP-9: cause an acute injury pattern in rats treated with bleomycin MMP-2, MMP-8, and MMP-9: play a role in the pathogenesis of ventilator-provoked lung damage.

MMP: matrix metalloproteinase; ASMC: airway smooth muscle cells; IGFs: insulin-like growth factors; IL: interleukin; IPF: idiopathic pulmonary fibrosis; TIMP: tissue inhibitor of metalloproteinases; CF: cystic fibrosis; FasL: Fas ligand; BAL: bronchoalveolar lavage; COPD: chronic obstructive pulmonary disease; LPS: lipopolysaccharide; UIP: usual interstitial pneumonia; PAF: platelet-activating factor; TNF: tumor necrosis factor; ALI: acute lung injury; RSV: respiratory syncytial virus; ARDS: acute respiratory distress syndrome; COVID: coronavirus disease; VILI: ventilator-induced lung injury; PI3-K: phosphatidylinositol 3-kinase; JNK: c-Jun N-terminal kinase; ERK: extracellular signal-regulated kinase; AP-1: activator protein 1; SP: surfactant protein; VEGF: vascular endothelial growth factor.

## Data Availability

Not applicable.
